# Peripheral manifestations of monocarboxylate transporter 8 deficiency: hepatic and skeletal muscle involvement

**DOI:** 10.3389/fped.2026.1978653

**Published:** 2026-09-11

**Authors:** Lina Doeoes, Alexander Laemmle

**Affiliations:** 1 Department of Pediatrics, Division of Pediatric Endocrinology, Diabetology and Metabolism, Inselspital, University Hospital of Bern, University of Bern, Bern, Switzerland; 2 University Institute of Clinical Chemistry, Inselspital, University Hospital of Bern, University of Bern, Bern, Switzerland

**Keywords:** hyperlactatemia, liver, monocarboxylate transporter 8 deficiency, skeletal muscle, thyrotoxicosis

## Abstract

Monocarboxylate transporter 8 (MCT8) deficiency, also known as Allan-Herndon-Dudley syndrome, is a rare X-linked disorder caused by pathogenic variants in the *SLC16A2* gene. It leads to impaired transport of thyroid hormones, particularly triiodothyronine (T3) and thyroxine (T4), across the blood-brain barrier and into neural cells. Consequently, the brain experiences reduced thyroid hormone availability, resulting in central hypothyroidism and severe neurodevelopmental impairment. In contrast, peripheral tissues remain exposed to elevated circulating T3, leading to chronic peripheral thyrotoxicosis. This review focuses on the effects of MCT8 deficiency beyond the neurological phenotype, with particular attention to liver involvement, skeletal muscle impairment, and hyperlactatemia. Current evidence suggests that peripheral organs are significantly affected by excess thyroid hormone signaling due to compensatory uptake through alternative thyroid hormone transporters. These alterations may contribute to metabolic disturbances and organ-specific manifestations that extend the clinical spectrum of the disease beyond the central nervous system. MCT8 deficiency should be considered a multisystem disorder rather than an isolated neurodevelopmental disease. Recognition of peripheral organ involvement may facilitate earlier diagnosis, improve clinical monitoring, and support the development of more targeted therapeutic strategies. Further research is needed to better understand the mechanisms underlying peripheral manifestations and their implications for patient care.

## 1 Introduction

MCT8 is a thyroid hormone transporter encoded by the *SLC16A2* gene located on the X-chromosome (1–5). It plays a crucial role in mediating the cellular uptake of thyroid hormones, particularly T3 and T4 (6–11). Furthermore, it contributes to the transport of these hormones across the blood-brain barrier (12–15). Thyroid hormones are essential regulators of numerous physiological processes, including neurodevelopment, metabolism, and organ function throughout life (16–25). Proper thyroid hormone signaling depends on a tightly regulated interplay between hormone transport (4, 12, 16), intracellular activation and inactivation via deiodinases 1 to 3 (DIO1–3) (26–30), and receptor-mediated genomic effects (31–33).

Pathogenic variants in the *SLC16A2* gene cause MCT8 deficiency, a rare X-linked disorder with an estimated prevalence of approximately 1 in 70’000 individuals (1, 2, 34). This condition is also referred to as Allan-Herndon-Dudley syndrome (AHDS), and was first described in 1944 (35). The condition is characterized by severe neurodevelopmental impairment, including intellectual disability, motor dysfunction, and reduced life expectancy (1, 6, 36). A hallmark of MCT8 deficiency is a distinct endocrine profile, characterized by central hypothyroidism due to impaired thyroid hormone transport into the brain, alongside peripheral thyrotoxicosis resulting from elevated circulating T3 levels (1, 36). Additionally, an increased T3/T4 ratio represents a characteristic biochemical finding in affected patients (2, 6, 37). While the central nervous system is deprived of thyroid hormones, peripheral tissues, such as the liver or skeletal muscle, remain exposed to excessive hormone activity, resulting in a complex and heterogeneous clinical phenotype (1, 38, 39).

Over the past decades, research has substantially improved the understanding of the genetic basis, endocrine characteristics, and neurological manifestations of MCT8 deficiency. In particular, studies have demonstrated the role of alternative thyroid hormone transporters and deiodinase activity in mediating peripheral thyrotoxicosis (16, 26–29, 38). Furthermore, recent therapeutic approaches, such as treatment with the thyroid hormone analogue tri-iodothyroacetic (TRIAC), have shown promising effects in reducing peripheral thyrotoxicosis and improving clinical outcomes (9, 34, 40–43).

However, despite these advances, several aspects of the disease remain insufficiently understood. In particular, the involvement of peripheral organs such as the liver and skeletal muscle, as well as the occurrence of metabolic alterations like hyperlactatemia, has not been systematically characterized. Existing studies addressing these features are limited in number, often heterogeneous in design, and frequently focus on related transporters rather than specifically on MCT8. Moreover, many findings are derived from animal models, which may not fully reflect human pathophysiology.

This represents a relevant gap in the current literature, as a deeper understanding of peripheral manifestations and metabolic disturbances in MCT8 deficiency could provide important insights into disease mechanisms and identify potential targets for therapeutic intervention.

Therefore, the aim of this literature review is to comprehensively assess the current literature on the involvement of liver function, skeletal muscle alterations, and hyperlactatemia in patients with MCT8 deficiency. By synthesizing the available literature, this review aims to contribute to a more integrated understanding of both central and peripheral aspects of the disease and to highlight directions for future research.

## 2 Materials and methods

This narrative literature review was conducted using the MEDLINE database accessed via the Ovid interface. MEDLINE was selected because it contains quality-controlled and indexed biomedical literature with Medical Subject Headings (MeSH) terms. To identify studies relevant to the aims of this review, searches were performed using the terms *MCT8 deficiency*, *hyperlactatemia*, *transaminase*, *liver*, and *skeletal muscle* (Supplementary Figure 1). For each term, both an exploded MeSH term search and a free-text search restricted to titles, abstracts, and keywords were conducted. Synonyms and term variations were included using Boolean operators and wildcards where appropriate.

The MeSH term and free-text searches for each concept were combined using the Boolean operator “OR”. To identify studies specifically addressing MCT8 deficiency, the MCT8 deficiency search was subsequently combined with each of the other search terms using the Boolean operator “AND”. The resulting searches were aggregated and screened for relevance (Supplementary Figure 1).

The initial MEDLINE search for *MCT8 deficiency* identified 483 publications and provided a broad overview of the available literature. To systematically address the aims of this review, a structured search strategy was subsequently applied using the predefined search terms *MCT8 deficiency*, *hyperlactatemia*, *transaminase*, *liver*, and *skeletal muscle*. Combined searches yielded 1,420 papers (Supplementary Figure 1).

To refine the search, studies were restricted to publications in English, human studies, and articles published after December 31, 2019, reducing the number of records to 232 (Supplementary Figure 1). Titles and abstracts were screened for relevance, and studies not primarily focusing on MCT8 deficiency or mainly addressing unrelated conditions or cancer-associated monocarboxylate transporters were excluded.

In addition, articles identified through the initial broad search and free-text searches were screened to ensure comprehensive coverage of the topic. The final selection consisted of studies addressing the pathophysiology and clinical manifestations of MCT8 deficiency, with particular emphasis on liver involvement, skeletal muscle impairment, hyperlactatemia, thyroid hormone transport, and emerging therapeutic approaches (Supplementary Figure 1).

## 3 Results

### 3.1 Thyroid hormone transporters

Thyroid hormones require specific transport proteins to cross cell membranes, since they cannot diffuse passively into cells (4, 31, 44, 45). This transport step is therefore essential for intracellular thyroid hormone action, particularly for reaching the nuclear thyroid hormone receptors (4, 17, 44, 46). In humans, a total of 16 transporters distributed across five major families are involved in thyroid hormone transport, forming a highly redundant and tissue-specific uptake system (4, 12) (Supplementary Table 1). These five families include organic anion transporting polypeptides (OATPs), L-type amino acid transporters (LATs), sodium/taurocholate cotransporting polypeptides (NTCPs), solute carriers (SLCs), and monocarboxylate transporters (MCTs).

Studies consistently reported marked heterogeneity in transporter expression between tissues (4, 12). Different organs express distinct combinations of transporters, which leads to variable dependence on individual systems (4, 12) (Supplementary Table 1). Most thyroid hormone transporters are not specific to thyroid hormones alone but also transport bile acids, amino acids, hormones, or pharmaceutical compounds (4). In contrast, MCT8 appears to be highly selective for thyroid hormones, with no confirmed alternative physiological substrates (4, 47). Transport mechanisms also differ between transporter families (4). While many transporters depend on ion gradients such as sodium co-transport or proton coupling, MCT8 operates via facilitated diffusion and is independent of both pH and electrochemical gradients (4).

### 3.2 Involvement of the liver in MCT8 deficiency

**Figure 1:**
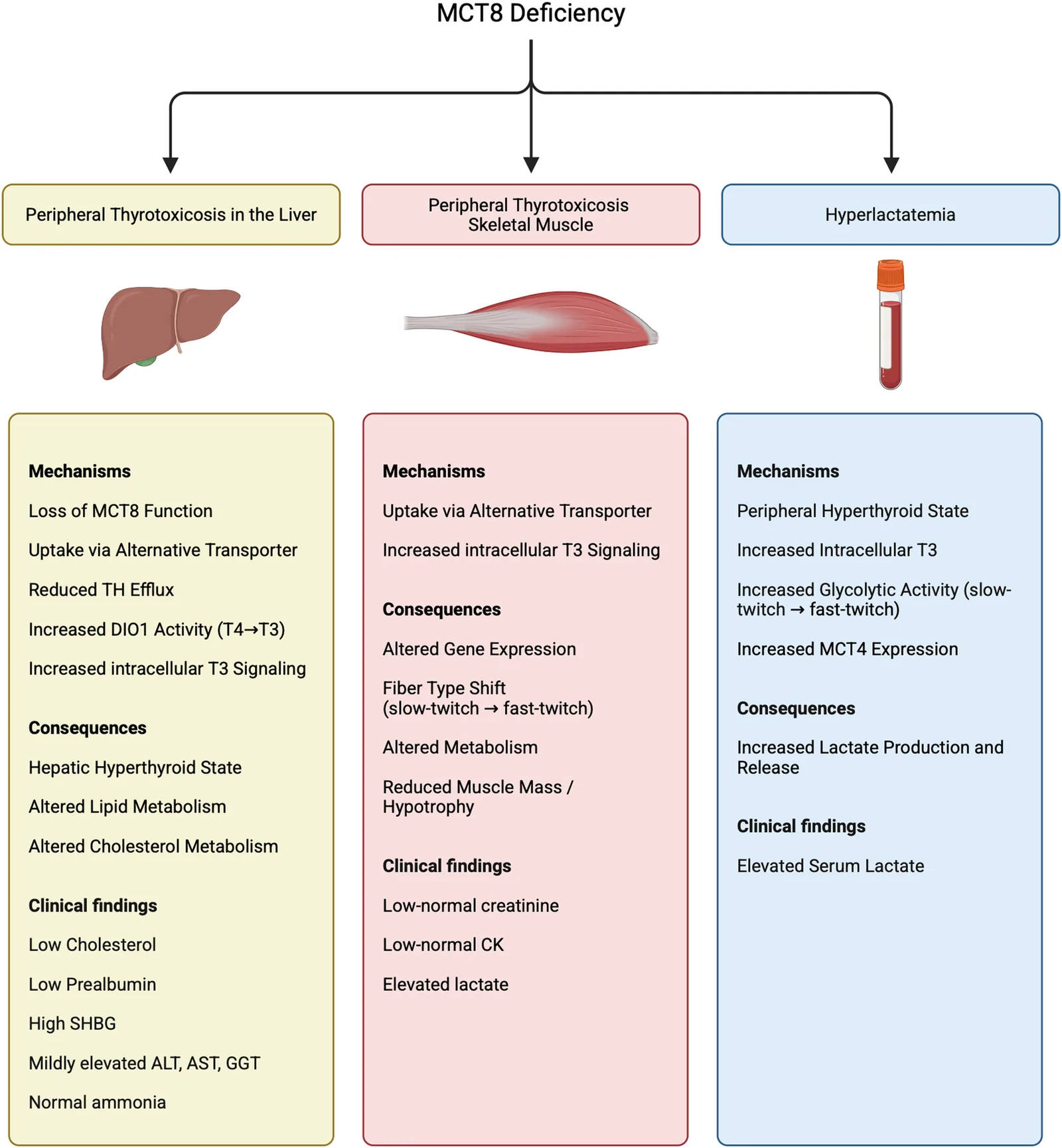
Peripheral manifestations of MCT8 deficiency. Current concepts regarding liver involvement, skeletal muscle alterations, and hyperlactatemia in MCT8 deficiency are summarized. Despite impaired MCT8 function, alternative thyroid hormone transporters enable increased intracellular T3 signaling in peripheral tissues, resulting in tissue-specific thyrotoxic effects and metabolic alterations. In the liver, this is associated with changes in lipid and cholesterol metabolism, whereas skeletal muscle shows altered gene expression, fiber type switching, and reduced muscle mass. Increased glycolytic activity and lactate release may contribute to elevated serum lactate concentrations observed in some patients with MCT8 deficiency. Figure generated using BioRender (https://biorender.com). alanine aminotransferase (ALT), aspartate aminotransferase (AST), sex hormone-binding globulin (SHBG), gamma-glutamyl transferase (GGT).

The liver is consistently identified as a major peripheral target of altered thyroid hormone signaling in MCT8 deficiency (3, 4, 6, 44) (Figure 1). Patients exhibit elevated hepatic thyroid hormone levels and increased thyroid hormone signaling in liver tissue (3, 4, 6, 17, 44). Studies reported that hepatocytes express multiple thyroid hormone transporters in addition to MCT8, allowing continued thyroid hormone uptake despite the loss of MCT8 function (4, 6, 8) (Figure 1). Reduced thyroid hormone efflux from hepatic tissue has also been described (4).

Evidence from Mct8 knockout mouse models demonstrated increased hepatic DIO1 activity, resulting in elevated intrahepatic T3 concentrations (3, 4, 48) (Figure 1). Clinically, patients frequently present with decreased serum cholesterol and prealbumin concentrations, elevated sex hormone-binding globulin (SHBG), and mildly increased alanine aminotransferase (ALT), aspartate aminotransferase (AST), and gamma-glutamyl transferase (GGT) levels (2, 4, 17, 49) (Figure 1). Ammonia concentrations are generally within the normal range (2, 4). Some patients have also experienced episodes of hepatic dysfunction following viral infections (2).

### 3.3 Involvement of the skeletal muscle in MCT8 deficiency

Skeletal muscle is profoundly affected in MCT8 deficiency (2–4, 50). Neuromuscular abnormalities are among the earliest manifestations and include global hypotonia, head lag, dystonia, hypertonia, spasticity, and persistent movement disorders (2, 3, 50). Most patients never acquire independent ambulation and remain wheelchair dependent (2, 3). Muscular hypotrophy, reduced muscle mass, impaired fine motor skills, and an underweight phenotype are also commonly reported (2, 3, 39).

Experimental studies demonstrated increased intracellular T3 concentrations in skeletal muscle of Mct8 knockout mice due to compensatory uptake through alternative thyroid hormone transporters (4, 8). Increased intracellular T3 was associated with altered thyroid hormone-dependent gene expression and a shift from slow-twitch oxidative fibers to fast-twitch glycolytic fibers (4, 16, 17) (Figure 1).

Biochemically, serum creatinine concentrations are typically within the low-normal range, while creatine kinase levels are generally normal but may increase after seizures or severe dystonic episodes (2, 4). Elevated serum lactate concentrations have also been reported in a considerable proportion of patients (6) (Figure 1).

### 3.4 Hyperlactatemia in MCT8 deficiency

Hyperlactatemia has been reported in a subset of patients with MCT8 deficiency (2, 6, 44). Groeneweg et al. observed mildly elevated lactate concentrations in three of eleven patients (6). In accordance, clinical data from one of our genetically confirmed MCT8-deficient patients revealed elevated lactate concentrations of up to 11 mmol/L (ref. < 1.8).

MCT8 itself does not transport lactate (4, 8, 12, 41). Lactate transport is mediated by other members of the monocarboxylate transporter family, particularly MCT1–4 (4, 8, 51, 52). Experimental studies further demonstrated increased Mct4 expression and upregulation of DIO2 in skeletal muscle of animal models with peripheral thyrotoxicosis (44) (Figure 1).

## 4 Discussion

### 4.1 Thyroid hormone transporters

The available evidence indicates that thyroid hormone transport is governed by a complex but partially redundant network (4, 12). However, this redundancy is unevenly distributed across tissues, creating selective vulnerability in organs that depend strongly on MCT8-mediated uptake, particularly the central nervous system (4, 12). In contrast, peripheral tissues such as the liver or skeletal muscle possess multiple alternative transporters that can compensate for the loss of MCT8 (4, 12).

### 4.2 Involvement of the liver in MCT8 deficiency

The findings suggest that alternative thyroid hormone transporters allow continued uptake of thyroid hormones into hepatocytes despite the absence of functional MCT8 (4, 6, 8). Together with reduced thyroid hormone efflux and increased local T3 production by DIO1, these mechanisms likely contribute to excessive intracellular thyroid hormone signaling and the development of a hepatic hyperthyroid state (3, 4, 48). The observed biochemical abnormalities therefore appear to reflect increased hepatic thyroid hormone action (3, 4, 6, 17, 44).

### 4.3 Involvement of the skeletal muscle in MCT8 deficiency

The available evidence indicates that skeletal muscle involvement in MCT8 deficiency is multifactorial (2, 3). While many clinical manifestations are likely secondary to severe neurological impairment, experimental studies demonstrate direct effects of peripheral thyroid hormone excess on skeletal muscle (2, 3). Compensatory thyroid hormone uptake maintains intracellular hormone availability but appears to promote excessive thyroid hormone signaling, altered gene expression, fiber type remodeling, and metabolic changes that may contribute to muscle pathology (4, 16, 17).

### 4.4 Hyperlactatemia in MCT8 deficiency

The available evidence suggests that hyperlactatemia is not a direct consequence of impaired MCT8 function but rather a secondary effect of altered thyroid hormone signaling and skeletal muscle metabolism. Increased MCT4 expression and enhanced local thyroid hormone activation may contribute to increased glycolytic activity and lactate production (44). However, the underlying mechanisms remain incompletely understood, and additional studies are required to clarify the pathophysiological basis of hyperlactatemia in MCT8 deficiency.

### 4.5 Conclusion and future perspectives

Taken together, the available evidence indicates that MCT8 deficiency is a complex multisystem disorder characterized by central hypothyroidism and peripheral thyrotoxicosis. Peripheral organs, particularly the liver and skeletal muscle, appear to be affected by altered thyroid hormone signaling, which may contribute to clinical manifestations such as elevated transaminases, muscle impairment, and hyperlactatemia. However, our literature review revealed that only limited studies are available regarding liver and skeletal muscle involvement and the mechanisms underlying hyperlactatemia in MCT8 deficiency. Most current knowledge is derived from relatively small patient cohorts and experimental animal models, limiting the understanding of tissue-specific disease mechanisms in humans.

**Figure 2:**
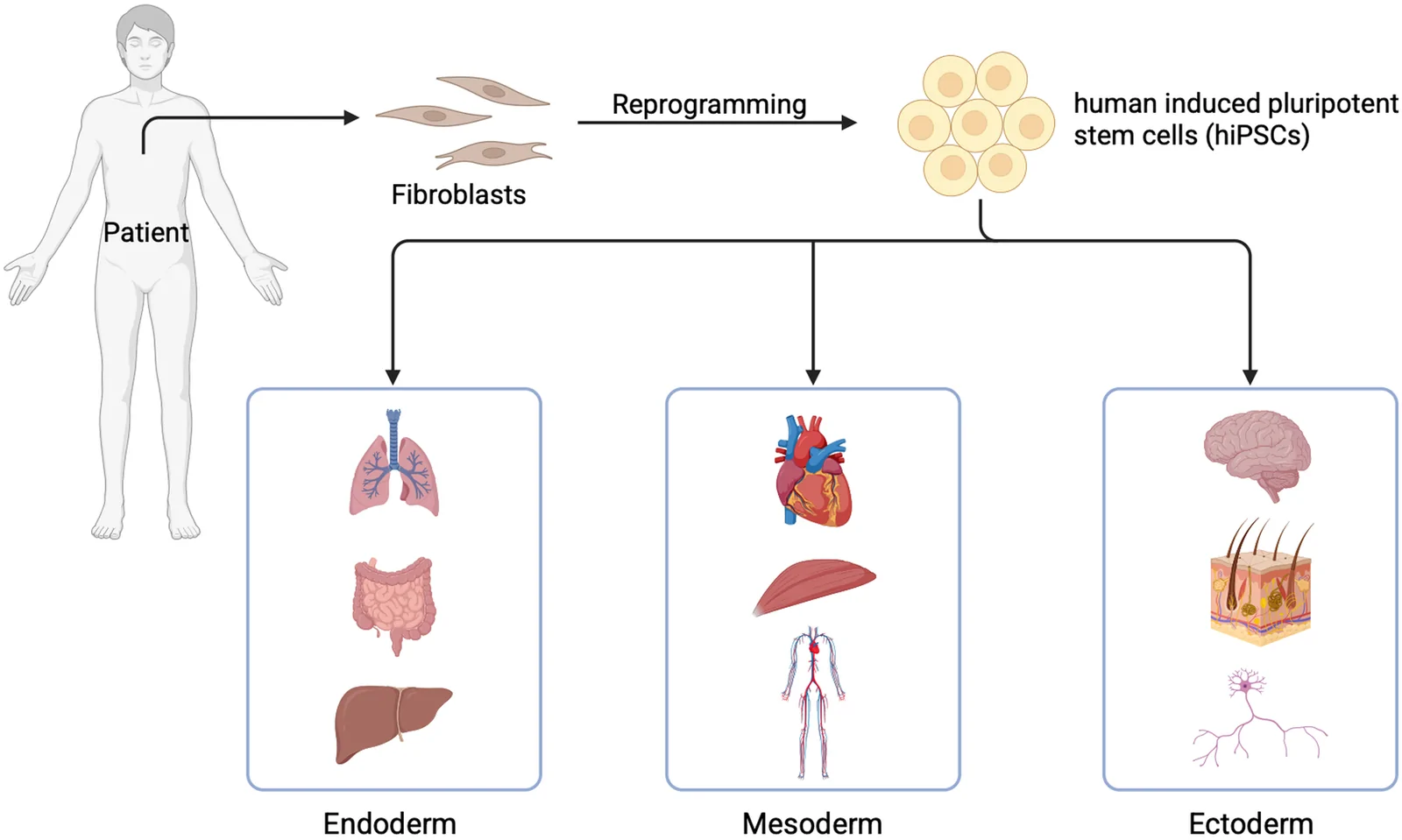
Generation of human induced pluripotent stem cells. This figure illustrates the process of generating patient-derived human induced pluripotent stem cells (hiPSCs) through cellular reprogramming of patient's fibroblasts. The generated hiPSCs have the ability to differentiate into all three germ layers. Figure generated using BioRender (https://biorender.com).

Therefore, in addition to preclinical animal models, future research should focus on human-based disease models. In particular, human induced pluripotent stem cell (hiPSC) technology offers a promising approach to investigate organ-specific pathophysiological processes in MCT8 deficiency. To study the peripheral effects of MCT8 deficiency on liver and skeletal muscle, hiPSC-derived hepatocytes and skeletal muscle cells from patients could reveal an interesting alternative approach to existing preclinical models (Figure 2) (53–58). Such MCT8 patient-derived *in vitro* models may not only improve our understanding of peripheral manifestations of the disease but also provide valuable platforms for drug testing and the development of targeted therapeutic strategies.
