# Supplementary material for: Peripheral manifestations of monocarboxylate transporter 8 deficiency: hepatic and skeletal muscle involvement
Source: Front Pediatr. 2026 Sep 11;14:1978653. doi: 10.3389/fped.2026.1978653 (PMC13612958; doi:10.3389/fped.2026.1978653)
Supplement: Supplementary file 1 [file Supplementaryfile1.docx]

Supplementary Material

# 1 Supplementary Figures and Tables

**Supplementary Figure 1.**	**Literature search and selection workflow**

Two searches were conducted using MEDLINE. First, a basic search for “MCT8 deficiency” was performed. Second, a Medical Subject Headings (MeSH) term and free-text search was conducted, combining “MCT8 deficiency” with additional search terms using the Boolean operator “AND”. Limits were applied to include only human studies, publications in English, and papers published from January 1, 2020 onward. Studies in which MCT8 was not the main focus, as well as articles addressing cancer or thyroid dysfunction in general, were excluded. The first search yielded 26 papers, while the second search identified eight papers, of which five were also identified in the first search. Figure generated using BioRender (<https://biorender.com>).

| **Thyroid Hormone Transporter Family** | **Transporters known** | **Location** |
| --- | --- | --- |
| **Organic Anion transporting Polypeptides** | OATP1A2, OATP1B1, OATP1B3, OATP1C1, OATP2B1, OATP3A1, OATP4A1, OATP4C1 | Brain, Kidney, Liver, Lung, Ciliar Body, Placenta, Testes, Adipose Tissue, Heart, Ovary, Gastrointestinal Tract |
| **L-Type Amino Acid Transporters** | LAT1, LAT2, LAT3, LAT4 | Brain, Placenta, Testes, Bone Marrow, Kidney, Spleen, Prostate, Ovary, Liver, Skeletal Muscle, Heart |
| **Sodium/Taurocholate cotransporting Polypeptides** | NTCP | Liver |
| **Solute Carriers** | SLC17A4 | Gastrointestinal Tract, liver, Pancreas, Kidney |
| **Monocarboxylate Transporters** | MCT8, MCT10 | Brain, Liver, Kidney, Thyroid, Pituitary, Skeletal Muscle, Heart, Hypothalamus |

**Supplementary Table 1.	Human thyroid hormone transporter families and their location.**

This table illustrates the five human thyroid hormone transporter families, their individual transporters, and human organs in which they are expressed.
